# Supportive care and osteopathic medicine in pediatric oncology: perspectives of current oncology clinicians, caregivers, and patients

**DOI:** 10.1007/s00520-020-05612-9

**Published:** 2020-07-09

**Authors:** Jennifer A. Belsky, Joseph Stanek, Micah A. Skeens, Cynthia A. Gerhardt, Melissa J. Rose

**Affiliations:** 1 Pediatric Hematology/Oncology/BMT, Nationwide Children’s Hospital, 700 Children’s Drive, Columbus, OH 43205 USA; 2 Departments of Pediatrics and Psychology, The Ohio State University, Columbus, OH USA; 3 The Center for Biobehavioral Health, Nationwide Children’s Hospital, Columbus, OH USA; 4 Department of Pediatrics, The Ohio State University College of Medicine, Columbus, OH USA

**Keywords:** Pediatric, Oncology, Osteopathic, Integrative medicine

## Abstract

**Background and objective:**

Many children receiving chemotherapy struggle with therapy-induced side effects. To date, there has been no literature investigating the needs, knowledge, or implementation of osteopathic manipulative treatments (OMT) as a supportive care option in pediatric oncology. We hypothesized that pediatric oncology clinicians, caregivers, and patients have (a) limited knowledge of OMT and (b) dissatisfaction with current supportive care options and (c) would be interested in having OMT available during chemotherapy, once educated.

**Methods:**

Participants included three cohorts: (1) children aged ≥ 9 years, diagnosed with cancer and actively receiving chemotherapy; (2) their caregivers; and (3) oncology clinicians at Nationwide Children’s Hospital. Participants completed 1:1 semi-structured interviews, which were audio-recorded, transcribed, and analyzed for thematic content regarding their perception of supportive care measures and views on OMT. Quantitative data was summarized descriptively.

**Results:**

A total of 60 participants completed the interview. Participants demonstrated limited awareness of osteopathic medicine; no participant had more than “some” knowledge of OMT. After education about OMT using a brief video, all clinicians, caregivers, and 95% of patients were receptive to OMT as a supportive care option. Major themes included the following: (a) patients have uncontrolled chemotherapy side effects, (b) improved supportive care options are desired, and (c) osteopathic medicine is a favorable supportive care adjunct.

**Conclusions:**

Pediatric oncology clinicians, caregivers, and patients reported a need for better management of chemotherapy-associated side effects and an interest in utilizing OMT. These findings support further investigation into the safety, feasibility, and efficacy of implementing OMT in the pediatric oncology clinical setting.

**Electronic supplementary material:**

The online version of this article (10.1007/s00520-020-05612-9) contains supplementary material, which is available to authorized users.

## Introduction

Survival rates for childhood cancer continue to rise, yet the side effects and long-term sequelae of anticancer chemotherapy remain an area of concern for clinicians, caregivers, and patients. Chemotherapy is the primary treatment for many types of pediatric cancers, and advancements continue to be made in the discovery of novel, more targeted, chemotherapy agents. Common therapy side effects can include pain, nausea, fatigue, constipation, and psychosocial struggles [1, 2]. Additional medications to combat side effects, unfortunately, each have their own side effect profiles [3]. In conjunction with pharmacologic therapies, supportive care therapies including physical therapy, complementary and alternative medicine (CAM), and massage therapy are utilized by children with cancer having side effects, with varying levels of success [4–6]. Studies show a need for continued investigation of therapies to prevent such side effects, improve symptom control, and optimize overall quality of life in children with cancer [7].

Osteopathic medicine is a distinct form of medical practice, defined by the National Center for Complementary and Integrative Health (NCCIH) as a “mind and body” complementary method that offers the added benefit of a hands-on approach to diagnoses and treatment of many medical conditions [8]. Doctor of Osteopathic (DO) physician training is identical to Medical Doctor (MD) physician training with the exception of some additional special focused classes. Osteopathic physicians receive 200 additional hours of dedicated training in the musculoskeletal system, nervous system, muscles, and bones. These types of physicians focus on disease prevention and strive to use hands-on techniques to help alleviate pain, restore motion, and influence the body to help function more efficiently. In addition to chemotherapy and medications, DO physicians have some hands-on techniques that can complement pharmaceuticals and surgeries. Although osteopathic medicine has been practiced since 1892 and an increasing number of physicians are being trained annually [9], there remains a lack of knowledge regarding this therapy. A large, single institutional hospital study surveying 474 employees showed that, while physicians in the hospital had the highest awareness, only 53.7% of respondents overall had any knowledge of osteopathic medicine [10]. There is limited research regarding knowledge gaps regarding osteopathic medicine in the oncology community among physicians, physician assistants, and advanced nurse practitioners. In addition, there have been no studies investigating caregiver and patient awareness of osteopathic medicine.

Osteopathic manipulative treatment (OMT) is one of the specialized therapeutic modalities used by DO physicians and incorporates techniques such as gentle stretching and manipulation to target specific muscle and nerve groups [11]. Studies have suggested that OMT has the potential to reduce constipation and pain [12–15] and decrease length of hospitalizations [12]; however, scarce literature evaluating the use of osteopathic medicine in either the adult or pediatric oncology disciplines exists. There are few studies addressing the safety and feasibility of utilizing OMT in the adult and pediatric populations, however no dedicated safety and feasibility studies in the pediatric oncology population [16, 17]. Given the need for improved therapies to combat unwanted side effects, combined with the paucity of osteopathic research, this study investigated clinician, caregiver, and patient knowledge of osteopathic medicine in the pediatric oncology population and explored the perception of possible utilization and barriers to OMT.

## Methods

### Participants

Following institutional review board approval, we recruited eligible clinicians and families from December 2018 to February 2019 at Nationwide Children’s Hospital. Inclusion criteria included (1) patients currently receiving chemotherapy, ≥ 9 years of age; (2) caregivers of an oncology patient receiving chemotherapy, 0–21 years of age; and (3) allopathic or osteopathic physicians or advanced nurse practitioners in the Division of Oncology at Nationwide Children’s Hospital. We excluded respondents unable to comprehend written English and participants unable to complete the study due to medical or learning disabilities. Total participants approached included 24 oncology clinicians, 21 pediatric oncology patients, and 20 caregivers of pediatric oncology patients. Of those approached, 20 clinicians, 20 caregivers, and 20 patients (including 6 patient/caregiver dyads) completed a semi-structured, qualitative interview in clinic.

### Procedures and measures

A single trained investigator (J.B.) provided participants a brief education on OMT which included a script detailing osteopathic medicine followed by a 1-min video demonstrating OMT on a child (Supplemental Material 1). Participants then completed 1:1 semi-structured qualitative interviews with open-ended questions and self-reported quantitative questions in REDCap. Participants were asked about the following: their experience with chemotherapy-induced side effects, how they have managed these side effects, how they felt OMT could potentially be utilized in the oncology setting, and any hesitancies about incorporating OMT into oncology care. Furthermore, clinicians and caregivers were asked how and when they felt OMT could be best introduced to their patient or child, respectively. Following the qualitative interview portion, participants were asked whether they would want to have OMT available as a supportive care option.

### Data analysis

All quantitative responses were stored securely in a REDCap database. Interviews were audio-recorded and stored using an encrypted Apple iPad. All interviews were conducted by the same trained investigator (J.B.) to ensure consistency. Participants were compensated $10 for their time. Basic participant demographic information, such as age and gender, was collected and entered into a de-identified database.

Digital recordings were transcribed verbatim by an external service. Using an iterative process, two investigators on the research team (J.B., J.S.) independently analyzed and coded interview transcriptions using constant comparison method [18]. Analysis was conducted by reading transcriptions in batches of five interviews at a time to gain an overview of the data. Major themes and subthemes were identified first from clinician transcripts, then caregivers, and lastly patients. This procedure allowed the investigators to continually examine if themes and codes held true or differed between participants. The researchers then collectively reviewed the initial coding scheme, and representative quotes of the themes were selected. Discrepancies were resolved by investigator discussion, and a final coding scheme was applied. Twenty interviews were conducted and analyzed in each group, with data saturation reached after the first 10 interviews [19]. To ensure the relevance and comprehensiveness of the results, a researcher (C.G.) experienced in qualitative data analysis and not involved in data collection reviewed the data collection plan, data samples, coding process, and outcomes to ensure that the findings accurately reflected all major themes. Interrater reliability was calculated between two of the initial coders (J.B. and J.S.) by identifying the number of times each comment was rated as fitting with one of the themes/subthemes. The proportion of agreement was 94% (kappa coefficient). Quantitative questions were summarized using descriptive statistics.

## Results

A total of 60 participants completed the interviews. Interviews lasted between 1.3 and 12.0 min (mean = 8.4 min). Twenty oncology clinicians participated (65% female), including 15 attending physicians and 5 nurse practitioners with a median of 7 years of clinical practice (SD = 6.8; range = 1–24). Twenty caregivers of pediatric oncology patients (65% female) with a median age of 40 years (range 27–71) and 20 pediatric oncology patients (30% female) with a median age of 17 years (range 10–28) completed the study. Leukemia (33%) and sarcoma (29%) were the most common cancer diagnoses, with a median of 15 years of age at diagnosis (range 9–21). Demographic characteristics are summarized in Table 1.

**Table 1:** Characteristics of participants

Demographics					
Providers		Patients		Caregiver	
Number of participants *n*	20	Number of participants *n*	20	Number of participants *n*	20
Male sex *n (%)*	7 (35)	Male sex *n (%)*	12 (60)	Male sex *n (%)*	4 (20)
Race *n (%)*		Race *n (%)*		Race *n (%)*	
White	17 (85)	White	18 (90)	White	19 (95)
Black	0 (0)	Black	2 (10)	Black	1 (5)
Other	3 (15)	Other	0 (0)	Other	0 (0)
Years of practice *median (range)*	7 (1–24)	Age (years) at diagnosis *median (range)*	15 (9–21)	Age (years) at survey *median (range)*	40 (27–71)
Oncology subspecialty *n (%)*		Type of cancer *n (%)*		Child’s cancer type *n (%)*	
Leukemia	4 (20)	Leukemia	6 (30)	Leukemia	8 (40)
Lymphoma	2 (10)	Lymphoma	3 (15)	Lymphoma	2 (10)
Embryonal	4 (20)	Sarcoma	9 (45)	Sarcoma	8 (40)
Sarcoma	4 (20)	Central nervous system	2 (10)	Neuroblastoma	1 (5)
Neuro-oncology	4 (20)			Central nervous system	1 (5)
General	2 (10)				
Title *n (%)*		Currently on therapy *n (%)*	19 (95)	Relationship to patient *n (%)*	
Medical doctorate	14 (70)	Age (years) at survey *median (range)*	17 (10–28)	Mother	14 (70)
Nurse practitioner	6 (30)			Father	4 (20)
				Other (grandparent, foster parent)	2 (10)

### Quantitative knowledge of osteopathic medicine

Although 100% (*n* = 20) of clinicians had heard of osteopathic medicine, only 50% (*n* = 20) of caregivers and 0% (*n* = 20) of patients had heard of osteopathic medicine. Responses on their knowledge varied from 70% of clinicians knowing “some” to 30% knowing “very little” or “none at all.” In contrast, 85% of caregivers and 100% of patients knew “very little” or “none at all” about osteopathic medicine. Neither clinician, caregiver, nor patient knew “a lot.” On quantitative assessment, after receiving the video and script education, 100% of clinicians, 100% of caregivers, and 95% of patients responded “yes” when asked if they were interested in having osteopathic medicine available as an adjunct supportive care treatment option (Table 2).

**Table 2:** Knowledge and receptiveness to osteopathic medicine

Question *n* (%)	Providers *N* = 20	Caregivers *N* = 20	Patients *N* = 20
1. Have you heard of osteopathic medicine?			
• Yes	20 (100)	10 (50)	0 (0)
2. How much do you feel you know about osteopathic medicine?			
• A lot	0 (0) 14	0 (0) 3	0 (0) 0
• Some	(70)	(15)	(0)
• Very little	5 (25)	7 (35)	0 (0)
• None at all	1 (5)	10 (50)	20 (100)
3. Would you want osteopathic medicine available as a treatment option?			
• Yes	20 (100)	20 (100)	19 (95)

### Qualitative themes

Thematic content analyses of transcribed interviews resulted in identification of three major themes that were consistently observed across all three participant groups: (a) pediatric oncology patients have uncontrolled chemotherapy side effects, (b) desire for better supportive care options, and (c) osteopathic medicine is a favorable supportive care option (Table 3).

**Table3:** Major themes and subthemes identified by participants

Common themes	% Identified		
	Providers *N* = 20	Caregivers *N* = 20	Patients *N* = 20
1. Patients have uncontrolled chemotherapy side effects	100%	100%	100%
• Frustration			
• Dissatisfaction			
• Poor symptom management			
2. Participants desire better supportive care options	95%	95%	80%
• Lack of alternative therapies			
• Non-pharmacologic interventions			
3. OMT is a favorable supportive care option	100%	100%	95%
• Beneficial intervention			
• Receptive			

*OMT* osteopathic manipulative treatment

#### Theme 1: Patients have uncontrolled chemotherapy side effects

Participants were interviewed regarding their experiences with chemotherapy and disease side effects. All three groups reported specific instances where chemotherapy side effects were difficult to manage. Most commonly, participants experienced poor symptom management particularly for nausea, vomiting, constipation, neuropathy, and pain. Vincristine was a major identified source of uncontrolled side effects, including constipation and neuropathy. Some clinicians further expressed that vincristine side effects often lead to unwanted dose reductions. Clinicians, caregivers, and patients expressed frustration with currently available treatments including insufficiency of multiple pharmacologic interventions and a lack of alternative available options (Table 4). One clinician specializing in solid tumors reflected on his experiences with trial-and-error stating, “We tried different cocktails and try to get the best regimen for them. But it’s sometimes a guessing process to which ones are going to work the best and at times we fail initially.” In addition, clinicians reported that families felt frustrated and hopeless and were open to alternative therapy options to control side effects. One physician said, “Parents felt like medicines worked nominally but weren’t working enough, it was taking a long time, and generally they were very frustrated. They kind of knew these side effects were potentially out there but didn’t really sense or appreciate the gravity or the significance of them in terms of their impact.”

**Table 4:** Participant perspectives major themes and quotes

Theme	Representative quote
Patients have uncontrolled chemotherapy side effects	1. Mother (child with leukemia): “We had nausea, a lot vomiting, definitely with methotrexate, especially with the high dose stuff. It just took all of her energy and all of her freedom.” 2. Physician (sarcoma): “I do not think they felt satisfied that we were able to address [uncontrolled side effects]. I would not say they were angry at us, but I think we talked through it and say basically ‘this is a difficult scenario where we do not want to do too many medications’. [This explanation] it falls short for our parents sometimes, especially in a child and a teenager.” 3. Physician (neuro-oncology): “He’s on everything that you could possibly think of and still vomits three to four times a day. We just kind of tell the family ‘well, that’s his baseline for now’, so, I do not think we have really figured out a way to manage him.” 4. Mother (child with leukemia): “She went weeks and weeks and weeks where I personally as her caregiver, 24 hour a day, had a timer that went of every 2 hours because she needed so many medications. So, she was miserable, and I was miserable.”
Participants desire better supportive care options 1. Lack of alternative therapies 2. Desire non-pharmacologic interventions	1. Nurse Practitioner (Leukemia): “I would love, love, love to be able to offer them more. But in my pocket right now I feel like those [medications] are my three best options.” 2. Patient (Sarcoma): “It’s just I wish I wasn’t on the pills. I wish there was a different way because I hate taking pills.” 3. Physician (Embryonal): “I mean in western medicine, we are really good at treating cancer. We’re not really good at treating the side effects of them so I think things like nausea, headaches, pain especially in our limb salvage patients who have a lot of musculoskeletal disruption over the course of treatment there’s probably opportunities where they could benefit from non-chemical means of treatment.”
Osteopathic medicine is a promising supportive care option 1. Beneficial intervention a. Low risk b. Noninvasive c. Non-pharmacologic 2. Receptive	1. Physician (leukemia): “I would be more than willing to give it a try since it’s not invasive and does not take long.” 2. Mother (child with leukemia): “I would be game with it if it would prevent her from having to take a pill that would snow her for 3 hours.” 3. Patient (leukemia): “It could definitely help. I think, given I’m not big on pills, that would definitely be a safer candidate.” 4. Physician (neuro-oncology): “None [hesitations] whatsoever. It would be exactly like physical therapy. For example, I do use physical therapy like they are an essential component of my specialty, brain tumors, and I would definitely incorporate an osteopathy doctor within our clinic and for my patients.” 5. Patient (leukemia): “No [hesitations], I mean I would love it.”

#### Theme 2: Desire for better supportive care options

All three cohorts expressed a need for better supportive care options to help control side effects. Participants expressed concern regarding a lack of available alternative supportive care therapies. Clinicians were often approached by patients and caregivers who specifically desired non-pharmacologic interventions to help with side effects and often felt clinician responses were inadequate. One clinician stated, “I’ve definitely had parents ask about more, or instead of medicine, is there something else we can try. I haven’t been able to give them a great answer.” Patients viewed taking pills as a burden and expressed a desire for non-pharmacologic interventions, with multiple patients having similar views to a 12-year-old patient who stated “It’s just I wish I wasn’t on the pills. I wish there was a different way, because I hate taking pills.”

#### Theme 3: OMT is a favorable supportive care option

Following brief description and education about OMT, participants discussed various ways OMT may be utilized for chemotherapy side effects. Clinicians and caregivers expressed a desire to consider OMT when patients experience side effects that are not well-managed with pharmacologic-based therapies. They perceived OMT as an intervention that could potentially benefit nausea, vomiting, constipation, neuropathy, or pain. Clinicians, caregivers, and patients viewed OMT as an attractive therapy with the potential to be utilized at home and decrease as-needed medications. Some caregivers described the benefits of hands-on techniques, specifically the benefit of physical touch for healing.

While no major barriers were identified, participants elicited several smaller barriers before integrating OMT into their current supportive care plans. After education about OMT, clinicians uniformly stated that they had minimal to no hesitation of recommending or referring their patients for OMT, yet they sought further education, as to when best to refer and how to introduce OMT to families. Similarly, caregivers also stated they would like printed or video educational materials describing OMT techniques. One clinician and one caregiver expressed concern about the availability of an osteopathic physician to perform treatments.

Participants were asked about their perceptions of the osteopathic techniques, specifically if they had any fears or hesitations. After watching the scripted video, caregivers and patients did not report any fears or hesitations. One patient stated, “Nothing scares me about OMT, I would love it.” Two patients expressed not wanting to be touched when they were ill, but one stated that if OMT were helpful, they would be willing to tolerate the physical touch. Other clinician’s comments centered on the need for additional research on osteopathic medicine in pediatric oncology patients to demonstrate efficacy and the feasibility of integration into clinic.

Clinicians and caregivers agreed that introduction to osteopathic medicine should occur early in the chemotherapy course. Several clinicians and caregivers stated that osteopathic medicine should be one of the first supportive care options discussed with families when chemotherapy side effects are explained during the initial consent process. Other clinicians and caregivers had some concerns about families being overwhelmed at the time of diagnosis and suggested opting for an introduction shortly after diagnosis. Regardless, most believed that having OMT available and offered within the first few weeks of therapy, before side effects arise, would be optimal. Many caregivers expressed at the end of interviews a desire to start OMT immediately, with one mother of a child with leukemia stating, “I’d try it right now if it were an option.”

## Discussion

Using a mixed methods approach, we assessed current knowledge and perceptions of pediatric oncology clinicians, caregivers, and patients, regarding pediatric oncology supportive care and osteopathic medicine. In addition to confirming the universal desire for improvement in supportive care options, this study verified the relative lack of knowledge of OMT or its potential roles in supportive care. Most oncology clinicians had “some” to “very little” knowledge of osteopathic medicine, while caregivers had “very little” to “none at all,” and zero patients had knowledge of osteopathic medicine.

Current supportive care measures alone are frequently insufficient in treating pediatric chemotherapy side effects [20–22]. Participants acknowledged their most common uncontrolled symptoms including neuropathy, nausea, and constipation, which is congruent with previously reported findings regarding inadequately controlled side effects. Previous studies demonstrate increased compliance, adherence, and outcomes when chemotherapy side effects are controlled in children. Our findings strengthen the existing literature where patients, particularly those ≥ 9 years of age, do not like taking oral medications, leading to poor adherence to therapy [23]. Previous literature has demonstrated that approximately 50% of patients with chronic illness do not take oral medications as prescribed (i.e., the correct dose, time, day, and/or correct route) [24]. In conjunction with uncontrolled side effects, there are many non-pharmacologic, supportive care options that could potentially improve medication adherence [25, 26]; however, OMT has not yet been explored as a possible adjunctive therapeutic option. As our study participants shared frustration and dissatisfaction with available pharmacologic supportive care options, we recognize the use of CAM in pediatric patients continues to increase [27]. Although OMT is considered a “mind and body” practice by the NCCIH, OMT is often included as a CAM modality in international studies, where patients have reported its use as an adjunct supportive care therapy. In a 2015 study from Switzerland, 53% of pediatric oncology patients utilized some form of CAM, with 13% of respondents utilizing osteopathic medicine [28]. As oncology caregivers and patients increasingly use non-traditional therapy options, clinicians must be knowledgeable on these interventions.

Familiarity with osteopathic medicine was low in all participants, indicating a significant knowledge gap. Our findings demonstrated that pediatric clinicians had less knowledge of osteopathic medicine compared to publications from adult-based literature, where most physicians felt “extremely knowledgeable.” This could be due to few osteopathic physicians at the institution practicing osteopathic medicine. Additionally, this discrepancy may be the result of a general lack of OMT practiced by osteopathic physicians at large pediatric institutions compared to rural adult hospitals [10].

Current literature does not include a role for OMT as a supportive care option, as it has not been studied in the childhood cancer population. Our study begins to address this gap by demonstrating minimal to no hesitation for OMT integration in all three participant groups. After education, participants were receptive to OMT, perceived it to be a low-risk, non-pharmacologic intervention and expressed minimal hesitations to implementation. All oncology clinicians and caregivers were interested in having osteopathic medicine available to patients, and all but one patient were interested. This acceptance is necessary in order to move from pilot to efficacy studies, as this study acknowledges a yet unmet opportunity for further osteopathic research to establish its safety, feasibility, and efficacy within pediatric oncology.

While there were no major barriers or hesitations identified, participants recognized several minor concerns about integrating OMT into their current supportive care plans, although most of these concerns would be alleviated through OMT education and a logistical plan for billing and workflow. Depending on the institution and resources available, any DO that practices OMT is able to treat pediatric cancer patients. Recognizing that the feasibility of osteopathic medicine in pediatric patients has not been well studied, several institutions have dedicated osteopathic clinics or osteopathic clinicians that are skilled in performing simple osteopathic techniques, feasibly during a patient clinic visit. Billing for OMT is commonly coverage by insurance providers and for oncology patients can be bundled with their outpatient clinic visit or hospitalization [29]. The main hesitation was lack of osteopathic research within the field, which, as noted, lays the groundwork for future feasibility and efficacy supportive care clinical trials for OMT in pediatric oncology patients. Participant consensus was to introduce OMT early in the patient’s treatment course, ideally to proactively mitigate chemotherapy-induced side effects.

Despite promising findings, the authors acknowledge limitations of the study. This is a small sample, single institutional study which may not be representative of the wider pediatric oncology population. Nonetheless, we obtained perspectives from clinicians with a wide range of clinical experience and areas of oncology expertise, as well as patients and families that represented a variety of cancer diagnoses, ages, and duration of cancer treatment. Another limitation was that the lead investigator was an osteopathic physician which could lead to implicit bias from participants. To best negate this, all quantitative responses were blinded from the interviewer, and an iterative script was used for qualitative questioning. As with most qualitative research, there is the potential for observation bias, which was mitigated by having two researchers that independently code the data. Although there was not participant checking, rigor in coding was ensured by two independent coders with high interrater reliability. Utilizing a mixed methods approach to interview three different populations, including the clinicians who dictate and offer supportive care measures, as well as the patients and families who receive that care, is a strength of our study.

## Conclusions

This study investigates current knowledge and perspectives of pediatric oncology supportive care management and investigates the views of implementing OMT as an adjunctive treatment option. We identified a need from clinicians, patients, and their caregivers for better supportive care options and, after education, the desire to have osteopathic medicine available as an adjunct treatment. These findings support the need for future, scientifically rigorous clinical trials investigating the feasibility, safety, and efficacy of OMT as a non-pharmacologic adjunctive supportive care therapy option for childhood cancer patients.

## Electronic supplementary material

ESM 1 (PDF 81 kb)

ESM 2 (MP4 3821 kb)

ESM 3 (MP4 6305 kb)

ESM 4 (MP4 4283 kb)

## Data Availability

Not applicable.
