# Supplementary material for: Supportive care and osteopathic medicine in pediatric oncology: perspectives of current oncology clinicians, caregivers, and patients
Source: Support Care Cancer. 2020 Jul 9;29(2):1121–8. doi: 10.1007/s00520-020-05612-9 (PMC7767897; doi:10.1007/s00520-020-05612-9)
Supplement: Supplementary file 1 — (PDF 81 kb) [file 520_2020_5612_MOESM1_ESM.pdf]

Supportive Care and Osteopathic Medicine in Pediatric Oncology: A Mixed Methods Assessment

Authors: Jennifer A. Belsky, DO[1*], Joseph Stanek, MS[1], Micah A. Skeens, PhD[1], Cynthia A. Gerhardt, PhD[2, 3], Melissa J. Rose, DO[1, 4]

Affiliations: [1] Pediatric Hematology/Oncology/BMT, Nationwide Children's Hospital, Columbus, OH, USA, [2] Departments of Pediatrics and Psychology, The Ohio State University, Columbus, OH, USA, [3] The Center for Biobehavioral Health, Nationwide Children's Hospital, Columbus, OH, USA, [4] Department of Pediatrics, The Ohio State University College of Medicine, Columbus, OH, USA

Journal Name: Supportive Care in Cancer

Supplemental Material

Provider Survey

1. Have you ever heard of osteopathic medicine?
   a. Yes
   b. No
2. How much do you feel you know about osteopathic medicine
   a. A lot
   b. Some
   c. Very little
   d. None at All

Provider Script:

Doctors of Osteopathic Medicine, or DO physicians, are fully licensed physicians who practice in all areas of medicine and surgery. DO physician training is identical to MD physician training with the exception of some additional special focused classes. Osteopathic physicians receive dedicated training in the musculoskeletal system, nervous system, muscles and bones. These type of physicians focus on disease prevention and strive to use hands on techniques to help alleviate pain, restore motion and influence the body to help function more efficiently.
In addition to chemotherapy and medications, DO physicians have some hands on techniques that can complement pharmaceuticals and surgeries.

Treatments may include Osteopathic Manipulative Treatment or (OMT), a hands- on treatment used to diagnose illness and injury and encourage your body's natural tendency towards self-healing. These treatments include applying specific amounts of pressure to different regions of the body with the goals of: treating structural and tissue abnormalities, restore muscle and tissue balance, as well as promote the overall movement of blood flow throughout the body.
Children undergoing chemotherapy suffer from many side effects from their cancer and medications.

In numerous research studies on both children and adults, OMT has been used to help alleviate many different side effects associated diseases or medications that help treat these illnesses. Osteopathic medicine has not been explored to help treat harmful chemotherapy and radiation side effects. These are services offered with your appointment.

<u>Short video descriptions read aloud during video:</u>

OMT 1: This is a demonstration of a technique called paraspinal inhibition. This is a technique in which the fascia around the spine is softened with one's hand to decrease sympathetic output from the sympathetic ganglion residing in the paraspinal area. It can be done with gentle or firm pressure depending on patient preference. It can be performed anywhere from 30 seconds to 3 minutes depending on how hypertonic the tissue is.

OMT 2: This is a demonstration of a technique called the mesenteric lift. This technique focuses on decreasing congestion and restrictions in the abdominal region by gently lifting the mesentery from each quadrant. It is not painful and uses gentle to intermediate pressure. This technique takes no more than two minutes on average.

OMT 3: This is a demonstration of a technique called suboccipital release. This is a soft tissue technique putting gentle to intermediate pressure on the suboccipital aspect of the spine to reduce tension stemming from sympathetic reflex activity from organs innervated by the cervical sympathetic ganglia. This technique takes between 30-90 seconds.

Qualitative Questions:

1. Drawing on your experience with many patients and their struggles with chemotherapy and disease side effects, please describe a story or scenario where you feel a patient may have experienced chemotherapy side effects that you felt were not well controlled?
    a. What did you try in response to these symptoms? What was the result?
    b. How did the family feel about these interventions?
2. Now that you have heard a little about osteopathic medicine, how do you feel that this therapy may be utilized for your patient population?
3. How and when do you feel osteopathic medicine would be best introduced to families?
4. Imagine there is an osteopathic physician available to your patients, what hesitations would you have recommending or referring your patients to receive osteopathic treatments?
5. What other information would you find helpful for both yourself and other providers to feel comfortable making a referral or recommending this treatment option to families?

End with Quantitative

1. If data existed that benefited your patient population, would you be interested in having this treatment modality/option available for your patients?

Caregiver Survey

Quantitative Questions

1. Have you ever heard of osteopathic medicine?
   a. Yes
   b. No
2. How much do you feel you know about osteopathic medicine
   a. A lot
   b. Some
   c. Very little
   d. None at all

Short video descriptions read aloud during video:

Doctors of Osteopathic Medicine, or DO physicians, are fully licensed physicians who practice in all areas of medicine and surgery. DO physician training is identical to MD physician training with the exception of some additional special focused classes. Osteopathic physicians receive dedicated training in the musculoskeletal system, nervous system, muscles and bones. These type of physicians focus on disease prevention and strive to use hands on techniques to help alleviate pain, restore motion and influence the body to help function more efficiently. In addition to chemotherapy and medications, DO physicians have some hands on techniques that can complement pharmaceuticals and surgeries.

Treatments may include Osteopathic Manipulative Treatment or (OMT), a hands- on treatment used to diagnose illness and injury and encourage your body's natural tendency towards self-healing. These treatments include applying specific amounts of pressure to different regions of the body with the goals of: treating structural and tissue abnormalities, restore muscle and tissue balance, as well as promote the overall movement of blood flow throughout the body. Children undergoing chemotherapy suffer from many side effects from their cancer and medications.

In numerous research studies on both children and adults, OMT has been used to help alleviate many different side effects associated diseases or medications that help treat these illnesses. Osteopathic medicine has not been explored to help treat harmful chemotherapy and radiation side effects. These are services offered with your appointment.

Short video descriptions read aloud during video:

OMT 1: This is a demonstration of a technique called paraspinal inhibition. This is a technique in which the fascia (or a type of tissue that attaches, encloses, and separates muscle and other internal organs) around the spine is softened with one's hand to "calm down" the nerve endings that can be irritating to muscles. It can be done with gentle or firm pressure depending on patient

preference. It can be performed anywhere from 30 seconds to 3 minutes depending on how tight the muscle/tissue is.

OMT 2: This is a demonstration of a technique called the mesenteric lift. This technique focuses on decreasing congestion and restrictions in the abdominal region by gently lifting the mesentery (abdominal tissue) from each quadrant. It is not painful and uses gentle to intermediate pressure. This technique takes no more than two minutes on average.

OMT 3: This is a demonstration of a technique called suboccipital release. This is a soft tissue technique putting gentle to intermediate pressure on the suboccipital aspect of the spine (muscle at the base of your head/neck) to reduce tension stemming from nerves that can be irritating to that muscle. This technique takes between 30-90 seconds.

Qualitative Questions

1. Thinking back in your child's cancer journey, please describe a story or scenario where your child may have experienced chemotherapy side effects that you felt were not well controlled?
2. What did the medical team or you try in response to these symptoms? What was the result?
3. Now that you've heard a little about osteopathic medicine, how do you feel that this therapy could have been utilized for your child, if at all?
4. What would make it easier or harder to include osteopathic medicine into your child's treatment? (may need a cue… you described x side effect)
    i. That sounds like it was a barrier, what are things that would make it easier (or vice versa if they describe what would make it easier first)?
5. How and when do you feel osteopathic medicine would be best introduced to families?
6. What other information would be helpful for you or other families to know about this type of service?

End with Quantitative

1. Would you be interested in having this treatment modality/option available for your child throughout their chemotherapy treatment?

Patient Survey

1. Have you ever heard of osteopathic medicine?
    a. Yes
    b. No

2. How much do you feel you know about osteopathic medicine
    a. A lot
    b. Some
    c. Very little
    d. None at all

Patient Script:

Doctors of Osteopathic Medicine, or DO physicians, are fully licensed doctors who prescribe medicine and do surgeries. These doctors have the same training as all other doctors, but they have some extra classes to learn more about the bones and muscles of your body. These type of doctors focus on stopping illness and try to use their hands to help make your muscles and body feel better.

Treatments may include Osteopathic Manipulative Treatment or (OMT), a hands- on treatment used to diagnose illness and injury and encourage your body to get better. These treatments include touching with gentle pressure on some of your muscles and nerves to try to make them feel better.

No doctors have looked at seeing if OMT could be helpful to treat symptoms related to your chemotherapy. No extra office visits would be needed, the treatments would be done in your clinic room or hospital bed and would not cost your mom or dad any extra money.

Short video descriptions read aloud during video:

OMT 1: This is a demonstration of a technique called paraspinal inhibition. This is a technique in which the special tissue in your spine is softened with one’s hand to help relax your muscles. It is similar to a massage, but more focused. It can be done with gentle or firm pressure depending on how you feel. It typically takes less than a minute.

OMT 2: This is a demonstration of a technique called the mesenteric lift. This technique focuses on improving your stomach nerves and muscles by “freeing them” from the other organs and tissues. It is not painful and uses gentle to medium pressure depending on how you are feeling. This technique takes no more than two minutes on average.

OMT 3: This is a demonstration of a technique called suboccipital release. This is a soft tissue technique putting gentle to intermediate pressure on the muscles behind your head that can get tight when your body undergoes stress. This technique takes between 30-90 seconds.

Qualitative Questions

1. Some kids feel that they have had positive things happen to them as a result of their cancer experience. Some kids have had negative things happen to them, but most kids

have had both positive and negative things happen. Can you give some examples of what have been some negative side effects of your chemotherapy or cancer that have bothered you the most?

    a. What did you the doctors or you and your family do to try and make you feel better? Did it work?

2. Now that you've heard a little about this new type of treatment, how do you think that this type of therapy may have worked for you?
3. Based on what you've seen in the videos, what do you think you would like or dislike about this type of treatment?

End with Quantitative

1. Would you want this type of treatment available to you throughout your chemotherapy medicines?
